# PReS-FINAL-2209: MEFV gene mutations in central and south-eastern European countries

**DOI:** 10.1186/1546-0096-11-S2-P199

**Published:** 2013-12-05

**Authors:** D Perko, M Debeljak, N Toplak, A Sediva, T Dallos, M Harjaček, M Jelušič, G Ristić, B Dérfalvi, K Mironska, S Rusoniene, D Kuzmanovska, N Kurjane, M Bataneant, T Avčin

**Affiliations:** 1 Department of Allergology, Rheumatology and Clinical immunology, University Medical Center Ljubljana, Ljubljana, Slovenia; 2 Center for Medical Genetics, University Children's Hospital, University Medical Center Ljubljana, Ljubljana, Slovenia; 3 Department of Immunology, University Hospital Motol and 2nd Medical faculty, Prague, Czech Republic; 4 2nd Department of Paediatrics, Comenius University in Bratislava, Bratislava, Slovakia; 5 Rheumatology clinic, Children's Hospital Srebrnjak, Zagreb, Croatia; 6 Department of Pediatrics, Division of Pediatric Rheumatology and Immunology, University Hospital Centre Zagreb, Zagreb, Croatia; 7 Institute for Health Protection of Mother and Child 'Dr Vukan Cupic', New Belgrade, Serbia; 8 2nd Department of Paediatrics, Sammelweis University, Budapest, Hungary; 9 Division for Primary Immunodeficiences, Department of Pediatrics Immunology, University clinic for children diseases, Skopje, Macedonia, The Former Yugoslav Republic Of; 10 Children's Hospital, Affiliate of Vilnius University Hospital, Santariskiu Klinikos, Vilnius, Lithuania; 11 University Pediatric Clinic, Medical Faculty, Ss. Cyriland Methodius University, Skopje, Macedonia, The Former Yugoslav Republic Of; 12 Centre of Clinical Immunology, Stradina Clinical University Hospital, Riga, Latvia; 13 3rd Pediatric Clinic, University of Medicine and Pharmacy 'Victor Babes', Timisoara, Romania

## Introduction

Familial Mediterranean fever (FMF) is rarely reported in patients from central and south-eastern European countries (CSEE). The reason for this might be that the prevalence of FMF in CSEE is exceedingly low or that the disease is significantly under-recognized among local physicians. Moreover, genetic testing is not available in most of the countries in the region.

## Objectives

The aim of this study was to assess the frequency of *MEFV *gene mutations in periodic fever patients from CSEE countries.

## Methods

We analyzed clinical, laboratory and genetic data of *MEFV *gene of all periodic fever patients who were followed at the University Children's Hospital Ljubljana from the beginning of 2006 to the beginning of 2013. In addition, free genetic testing was provided for suspected FMF patients with periodic fevers from the countries of the CSEE region. Genetic testing was performed in the Genetic laboratory of the University Children's Hospital Ljubljana. All 10 exons and intron/exon regions of *MEFV *gene were directly sequenced with ABI Prism 310 Genetic analyzer.

## Results

In total, 156 periodic fever patients were tested for *MEFV *gene mutations; 118 from Slovenia, 14 from Czech Republic, 6 from Slovakia, 4 from Croatia, 4 from Romania, 3 from Macedonia, 2 from Serbia, 2 from Hungary, 2 from Latvia and 1 from Lithuania. 73% of the populations were children under the age of 18, mean age at diagnosis was 6.6 years. 27% were adult, mean age at diagnosis was 46.4 years. 53% of patients were female and 47% were male.

31 patients (20%) were found to have at least one mutation. 22 patients have had one mutation only; Slovenia 9/15, Czech Republic 7/8, Slovakia 1/3, Macedonia 2/2, Latvia 1/1, Hungary 1/1 and Croatia 1/1. 8 patients have had two mutations; Slovenia 6/15, Slovakia 1/3, Czech Republic 1/8 and 1 patient from Slovakia has had 3 mutations. Homozygous mutation was found only in one patient from Czech Republic. 1 novel *MEVF *gene mutation was identified (S730F) in patient from Slovenia.

12 different mutations were found. The 2 most frequently found were M694V (27%) and K695R (22%), followed by P369S (12%), R408Q (12%), I591T (7%), E148Q (5%), E167D (2%), A289V (2%), F479L (2%), V726A (2%), S730F (2%) and A744S (2%).

## Conclusion

*MEFV *gene mutations were identified in 31/156 (20%) patients with periodic fevers from CSEE countries. In order to increase the number of positive results of *MEFV *genetic testing clinical criteria for FMF diagnosis should be followed. We suspect that clinical manifestations of FMF could be influenced by the regional environment. We are planning to evaluate genotype-phenotype correlation in *MEFV *mutation positive patients in CSEE countries in the future.

## Disclosure of interest

None declared.

